# Draft Genome of the Edible Oriental Insect *Protaetia brevitarsis seulensis*

**DOI:** 10.3389/fgene.2020.593994

**Published:** 2021-01-13

**Authors:** Joon Ha Lee, Myunghee Jung, Younhee Shin, Sathiyamoorthy Subramaniyam, In-Woo Kim, Minchul Seo, Mi-Ae Kim, Seong Hyun Kim, Jihye Hwang, Eun Hwa Choi, Ui Wook Hwang, Jae Sam Hwang

**Affiliations:** ^1^Department of Agricultural Biology, National Institute of Agricultural Sciences, Rural Development Administration, Wanju, South Korea; ^2^Research and Development Center, Insilicogen Inc., Yongin, South Korea; ^3^Department of Biology Education, Teachers College and Institute for Phylogenomics and Evolution, Kyungpook National University, Daegu, South Korea

**Keywords:** Cetoniinae, Kolbe, genome, *Protaetia brevitarsis seulensis*, edible insect

## Introduction

Insects hold the template for significant technological and biological inventions, since most of them are smaller in size and have different characteristics. It could help human lives, if scientists mimic their characteristics for sensors, robotics, agriculture, and medicine. Recently, insects were identified as an alternative source for meat to meet the Food and Agricultural Organization (FAO) food demand for the growing population, which is estimated to be 9 billion by 2050 (Han et al., 2017). The recent progress and research interest in the field of entomophagy explain the importance of insect breeding (Raheem et al., 2019). In parallel, the inherited problem in the selection of insects for breeding is also harmful to the environment. To cite an example, *Locust*, a grasshopper group rich in nutrients and protein content, can be utilized as a substitute for meat, but it is highly harmful to the environment and food crops worldwide (Le Gall et al., 2019). Hence, it is essential to carefully adapt an insect from the indigenous population around the world, i.e., people who consume insects in their regular diet for various reasons. Moreover, insect breeding is estimated to reduce CO_2_ emission in the atmosphere (i.e., up to 18%) when compared to animal breeding, which is as crucial as food production (Raheem et al., 2019). Other major drawbacks of insect-based foods are toxicities and allergens, which need to be eliminated through detailed characterizations. By considering all these factors, the genetic make-up was initiated through a large insect genome project to fuel detailed characterizations (i.e., i5K insect genomes). However, for various reasons, the project has not reached the desired goal so far (Li et al., 2019a). Furthermore, the highest-sequenced species in i5k belong to the Coleoptera taxonomical order, which has more edible insects with beneficial medicinal and agricultural importance.

In South Korea, the estimated market value for edible insects in 2020 is USD 457 million (Han et al., 2017). Notably, the white-spotted flower chafer beetle has contributed to the highest revenue among other edible insects. As per the Korean Ministry of Agriculture, Food and Rural Affairs (https://www.mafra.go.kr/english/1412/subview.do) report, the insect breeding industries rose from 726 in 2015 to 2,318 (~300%) in 2018. Based on this knowledge, we selected the oriental edible beetle insect, *Protaetia brevitarsis seulensis*, also known as Kolbe, for genome sequencing (referred to as Kolbe in the rest of the article). Kolbe belongs to the Cetoniinae family, widely used in oriental medicine to treat various diseases. Also, it was approved temporarily as a food material by the Ministry of Food and Drug Safety of Korea (MFDS) in 2014 (Lee et al., 2017b). It has been highly suggested for use in cookies and cosmetics (Lee et al., 2017a). Therapeutic components such as phenols (Kim et al., 2020), alkaloids (Lee et al., 2017a), fatty acids (Li et al., 2019b), and bio-active peptides (Lee et al., 2016) were characterized from this species to treat different diseases. In agriculture, the waste management process, such as livestock manure processing (Yin et al., 2018) and plant cellulose decomposition, uses Kolbe (Li et al., 2019b). However, in the genus *Protaetia*, there are only two species, namely, *Protaetia brevitarsis* and Kolbe. The draft genome of *Protaetia brevitarsis* habituated in China, as reported so far, is heterozygous. But as per our knowledge, this is the first draft genome of the species Kolbe widely present in South Korea.

### Value of the Data

The Kolbe draft genome is a base/reference for all the molecular studies in the *Protaetia* genus. It could be a valuable resource to conduct a comparative analysis among the species in the genome of the *Protaetia* genus to enhance breeding.

## Materials and Methods

### Insect Sample Collection

Kolbe was maintained in the insect rearing facility of the National Institute of Agricultural Sciences (Wanju, Republic of Korea). The larvae and adults were reared on fermented oak sawdust in a constant rearing room at 25°C ± 1°C, under 50–60% relative humidity (RH) and a 14 h light: 10 h dark photoperiod cycle.

### DNA and RNA Preparation for Sequencing

Eighteen individual last instar larvae of Kolbe were selected for DNA sample extraction from the whole body for the genomic sequencing. For the genomic DNA isolation, the sample was washed with PBS, sterilized with 70% ethanol, and then anesthetized on ice. The entire body was fixed, and the dissected integument was then cut along the ventral part, and the guts were removed. The carcass was then quickly ground in liquid nitrogen using a mortar and pestle. The ground tissues were used for genomic DNA isolation using a Wizard Genomic DNA Purification Kit (Promega, USA) according to the manufacturer's instruction. The quality and quantity of the DNA sample were examined using ultraviolet (UV) absorbance and gel electrophoreses. Additionally, total RNA from four different tissues (fat body, gut, muscle, and hemocytes) and four different developmental stages (egg, larva, pupa, and adult) were isolated for whole transcriptome sequencing. Briefly, each tissue (fat body, gut, and muscle) was collected from three individual last instar larvae after dissection, as mentioned above. For the collection of hemocytes, three individual last instar larval hemolymph were directly collected into sterile tubes containing anticoagulant buffer (62 mM NaCl, 100 mM glucose, 10 mM EDTA, 30 mM Sodium citrate, 26 mM citric acid, and pH 4.6) on ice in triplicate, and they were centrifuged for 10 min at 1,000 g at 4°C to remove the supernatant. The tissues were homogenized in a 1.5 ml tube containing TRIzol reagent (Invitrogen, Carlsbad, CA, USA) using a pestle. In the case of the developmental stage sample, each stage of the three individual samples was washed with 70% ethanol to reduce microbial contamination from its surface. After ethanol volatilization, the individual was then quickly ground into a fine powder in liquid nitrogen, except for the eggs. For the extraction of eggs, 10 eggs were homogenized in a 1.5 ml tube containing TRIzol reagent using a pestle, in triplicate. RNA quantitation was performed by UV absorbance, and gel electrophoreses further confirmed its quality.

### Genome Size Estimation and Assembly

The isolated DNAs were sequenced using two different sequencing methods, i.e., Pacific bioscience (PacBio, Sequel), and Illumina (NextSeq500), which is familiar for long and short read sequencing. DNALink, the authorized service provider in South Korea, conducted complete experimental procedures. The Illumina paired-end sequences were initially subjected to the filtering of technical artifacts (i.e., base calling error [PHERD quality score (*Q* ≤ 20)], and adapters using Trimmomatic-0.32 method (Bolger et al., 2014). Finally, the genome size estimation was carried out using the *k*-mer-based method with the Jellyfish v2.0 by calculating the genome coverage depth and size, as explained in the Sea Bream genome article (Shin et al., 2018). Additionally, these Illumina reads were used for the error correction of PacBio reads with clc-assembly-cell v5.1.1.184548-201811011136. Finally, the corrected PacBio reads were used for the initial draft version of the Kolbe genome with FALCON-Unzip v0.30 and haplotype assembler (Chin et al., 2016). The assembled contigs were assessed for completeness using the BUSCO v3.0, with the insecta_odb9 reference datasets (Waterhouse et al., 2017).

## Repeat Regions Prediction and Classification

The repeat regions in Kolbe were predicted using RepeatModeler (www.repeatmasker.org/RepeatModeler/) and classified into subclasses with the reference Repbase v20.08 database (www.girinst.org/repbase/) (Bao et al., 2015). Finally, the repeats were masked in the genome using RepeatMasker v4.0.5 (www.repeatmasker.org) with RMBlastn v2.2.27+.

## Gene Prediction and Annotation

The genes from the Kolbe draft were predicted using an in-house gene prediction pipeline. It includes three modules: an evidence-based gene modeler (EVM), an ab-initio gene modeler, and a consensus gene modeler. The transcriptomes from the two methods [i.e., Illumina (132.8 Gb) and IsoSeq (0.7 Gb)] were mapped to the Kolbe repeat masked draft genome using TopHat, and Cufflink (Trapnell et al., 2012) and PASA (Haas et al., 2003) marked the transcripts and gene structural boundaries respectively. The *ab-initio* gene modeler and EVM (includes Exonerate (Slater and Birney, 2005), AUGUSTUS (Stanke et al., 2006), and GENEID (Blanco et al., 2002)) were trained with several genomes. The final gene and transcript models were optimized with a consensus gene modeler with EVidenceModeler (Haas et al., 2008). The functional annotations (i.e., gene ontologies (GO), KEGG Pathways) for the final model were obtained from the Blast2GO method (Götz et al., 2008).

## Comparative Genome Analysis

The total genes of Kolbe were subjected to orthologous analysis to observe the insights of protein compositions among other insects in the Coleoptera taxonomical order. Seventeen genomes (including Kolbe) from fifteen families were used in the ortholog analysis using the OrthoMCL method (Li et al., 2003) along with three databases, i.e., cytochrome P450 engineering database (CYPED), carbohydrate-active enzymes database (CAZY) and KEGG database, to obtain the functions (Table 1E). The single-copy genes from the given genomes were subjected to Bayesian evolutionary analysis sampling trees (BEAST), phylogenetic tree reconstruction method, to assess evolutionary time and similarity position among the given genomes (Suchard et al., 2018). Furthermore, to determine the gain and loss of the genes in the given genomes, the proteins were subjected to CAFE v3.1 (Han et al., 2013) method.

**Table 1:** Summary of the sequencing till annotation of *Protaetia brevitarsis seulensis* draft genome.

**Types**	**PacBio(Gb)**	**Illumina(Gb)**
**(A) SEQUENCING**		
DNA	31.1	277.7
RNA	0.7	132.8
**(B) ASSEMBLY**		
Estimated genome size (bp)	656,797,776	
Contigs	224	
Contig length (bp)	692,712,625	
Average length (bp)	3,092,467.08	
Minimum length (bp)	26,261	
Maximum length (bp)	16,895,244	
N50 (bp)	4,997,170	
NG50 (bp)	5,158,302	
N (%)	0	
GC (%)	33.42	
Repeat (%)	344,334,720(49.71%)	
BUSCO (insecta) complete (%)	99.03	
**(C) STRUCTURAL ANNOTATIONS**		
# of genes	23,551	
Average gene length (bp)	8,217.32	
Gene coverage (%)	193,526,041 (27.94%)	
GC in CDS (%)	43.67	
Exon/Gene	3.97	
Average exon length (bp)	250.25	
Exon coverage (%)	23,416,675 (3.38%)	
Average intron length (bp)	2,429.34	
Intron coverage (%)	170,109,366 (24.56%)	
**(D) FUNCTIONAL ANNOTATIONS**		
No hits	7,884 (33.48%)	
Blast hits	15,667 (66.52%)	
GO	10,844 (46.04%)	
KEGG	8,474 (35.98%)	
COG	8,565 (36.37%)	
PfAM	10,821 (45.95%)	
SignalP	1,553 (6.59%)	
TmHMM	3,227 (13.7%)	
**(E) SPECIES COMPARISON**		
Secondary metabolite pathways(KEGG)	182	
Fatty acid metabolisms(FATT)	179	
Cytochromes(CYP)	15	
Carbohydrate-Active enZYmes(CAZY)	221	

## Preliminary Analysis Report

**Figure 1:**
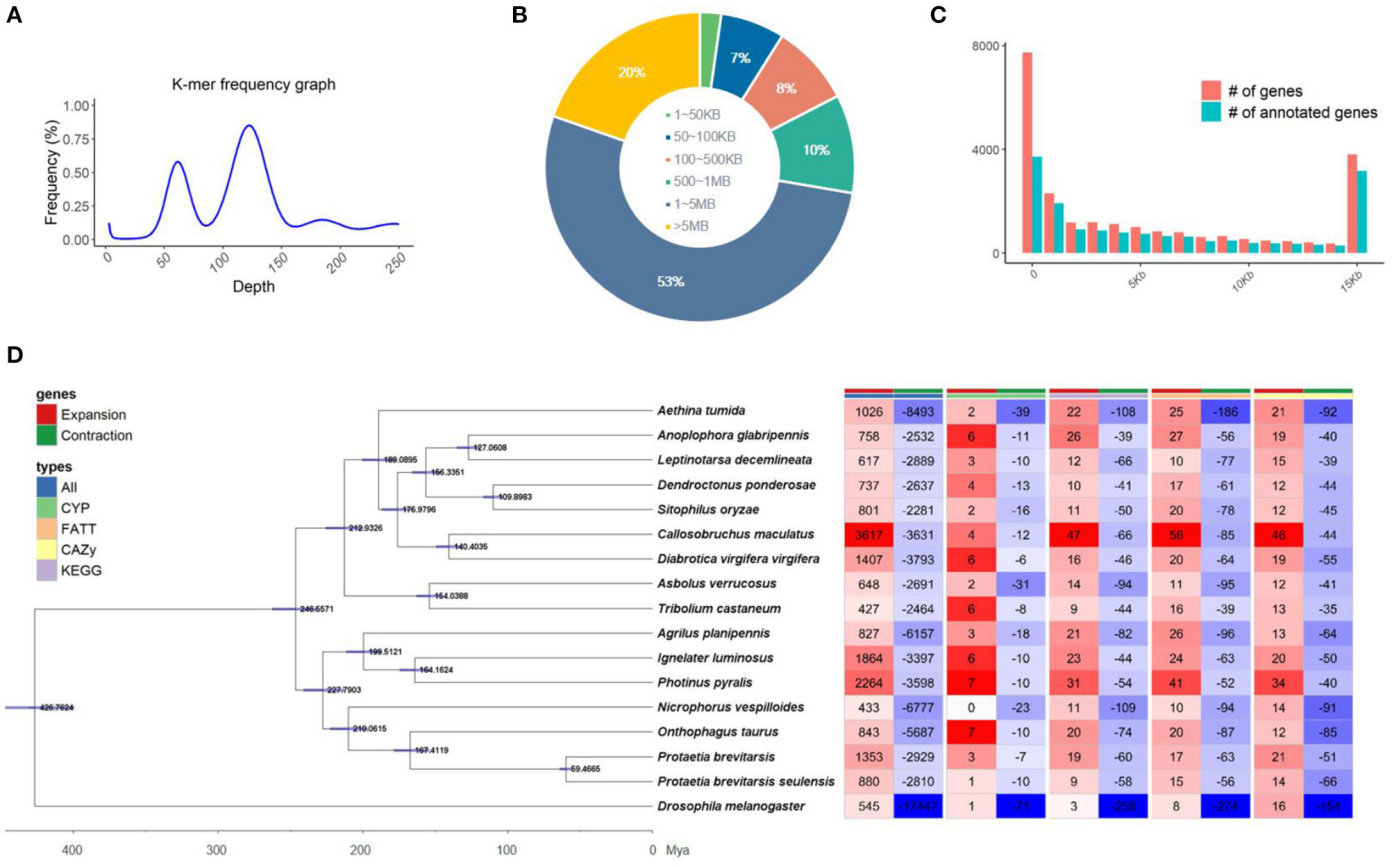
Summary of the sequencing; **(A)** Genome size estimation, **(B)** Contig length distribution, **(C)** Genes with annotation overview, **(D)** Gene expansion and contraction among the insect Coleoptera order genomes along with the phylogenetic tree reconstructed by BEAST with single-copy genes.

Initially, the genome size of Kolbe was estimated to be 656.8 MB, with 277.7 GB (401X) of short-read sequences (Figure 1A). The 692.7 MB of the representative draft genome was assembled into 224 contigs from 31.1 GB (45X) of error-corrected long read sequences (Table 1A; Figures 1B,C). The N50 of the assembled genome is 4.9 MB bases, and 344 MB of the assembled contigs were covered by repeats, which are unclassified elements. Totally, 23,551 genes were predicted from the genome with an average size of 8,217.3 bases, with the BUSCO score for completeness being 99% (Table 1B,C). A total of 15,667 (66.52%) genes are known to have homologous sequences in GenBank, and 10,844 (i.e., 46.04%) genes also have their gene ontology descriptions (Table 1D and Figure 1C). The evolutionary relationship among these genomes was assessed with 218 single-copy genes through phylogenetic tree reconstruction. The genomes were grouped into exact family clans without any distortion. In continuation, the gain and loss among those genomes were also assessed for the Kolbe genome (Figure 1D). Additionally, the cytochrome family genes from tissue-specific, stage-specific, and differential assessments were conducted. Among these, the Halloween family genes were observed to have differential and tissue-specific expression, which is involved in insect hormone biosynthesis (Rewitz et al., 2007). The specific and differential expressions were observed from RNA-Seq, and the detailed expressions are given in the additional file (Additional File 1).

## Data Availability Statement

The complete sequences generated in this study was deposited to the SRA repository under the accession PRJNA648262. The assembled contigs and its annotation files (CDS, gff, repeats, and proteins) are available in figshare: https://figshare.com/s/5e095ac1bf7a63411d23) repository with all the annotations details in Readme file.

## Author Contributions

JL, MJ, SS, and YS: genome assembly and annotations. MJ, SS, and YS: manuscript preparation. I-WK, MS, M-AK, SK, JH, EC, and UH: sampling and sequencing. JSH: funding and modeling the study. All authors contributed to the article and approved the submitted version.

## Conflict of Interest

MJ, SS, and YS were employed by the company Insilicogen Inc. The remaining authors declare that the research was conducted in the absence of any commercial or financial relationships that could be construed as a potential conflict of interest.
