# Supplementary material for: Draft Genome of the Edible Oriental Insect Protaetia brevitarsis seulensis
Source: Front Genet. 2021 Jan 13;11:593994. doi: 10.3389/fgene.2020.593994 (PMC7838600; doi:10.3389/fgene.2020.593994)
Supplement: Supplementary file 1 [file Data_Sheet_1.PDF]

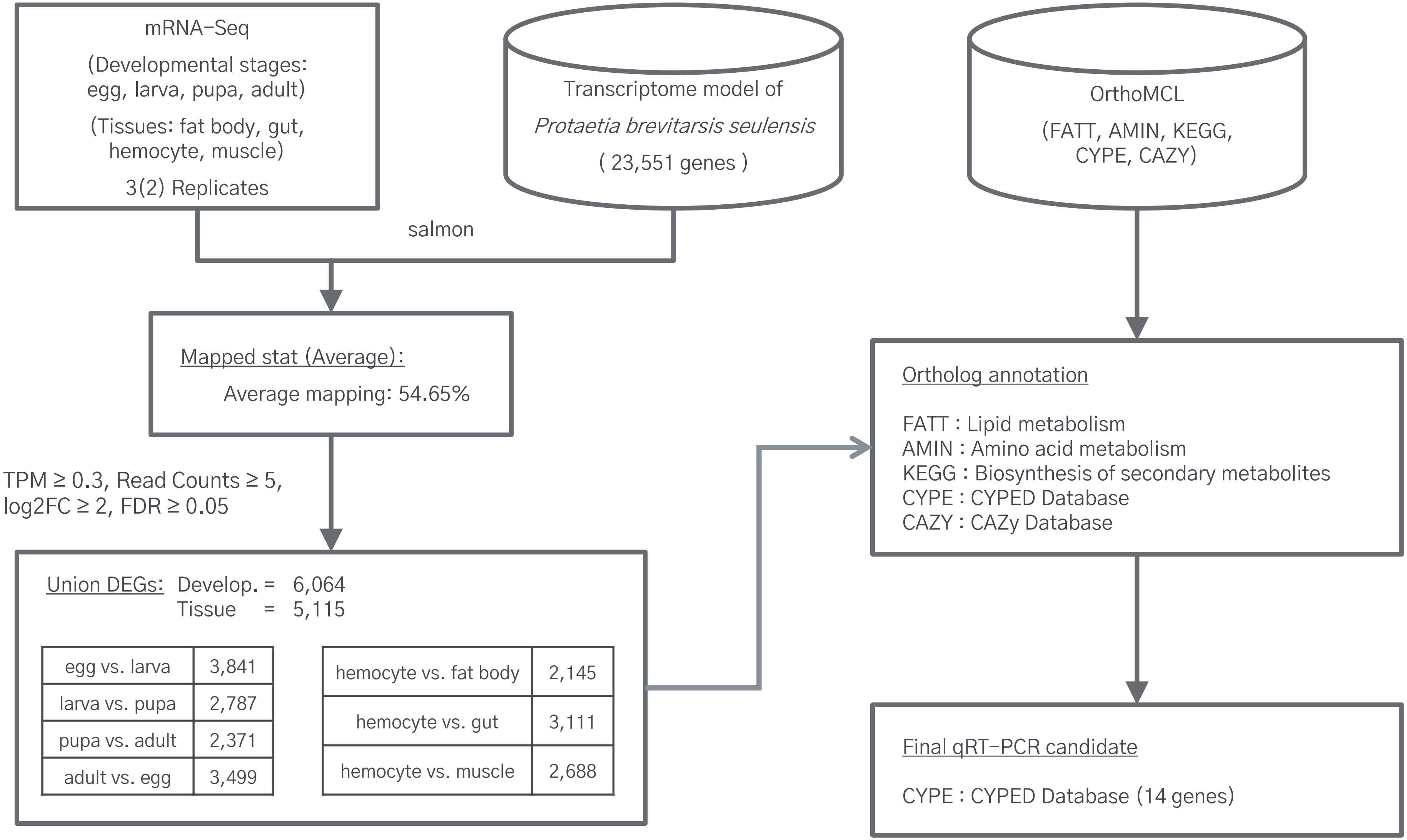

The work flow overview of the RNA_Seq expression analysis.

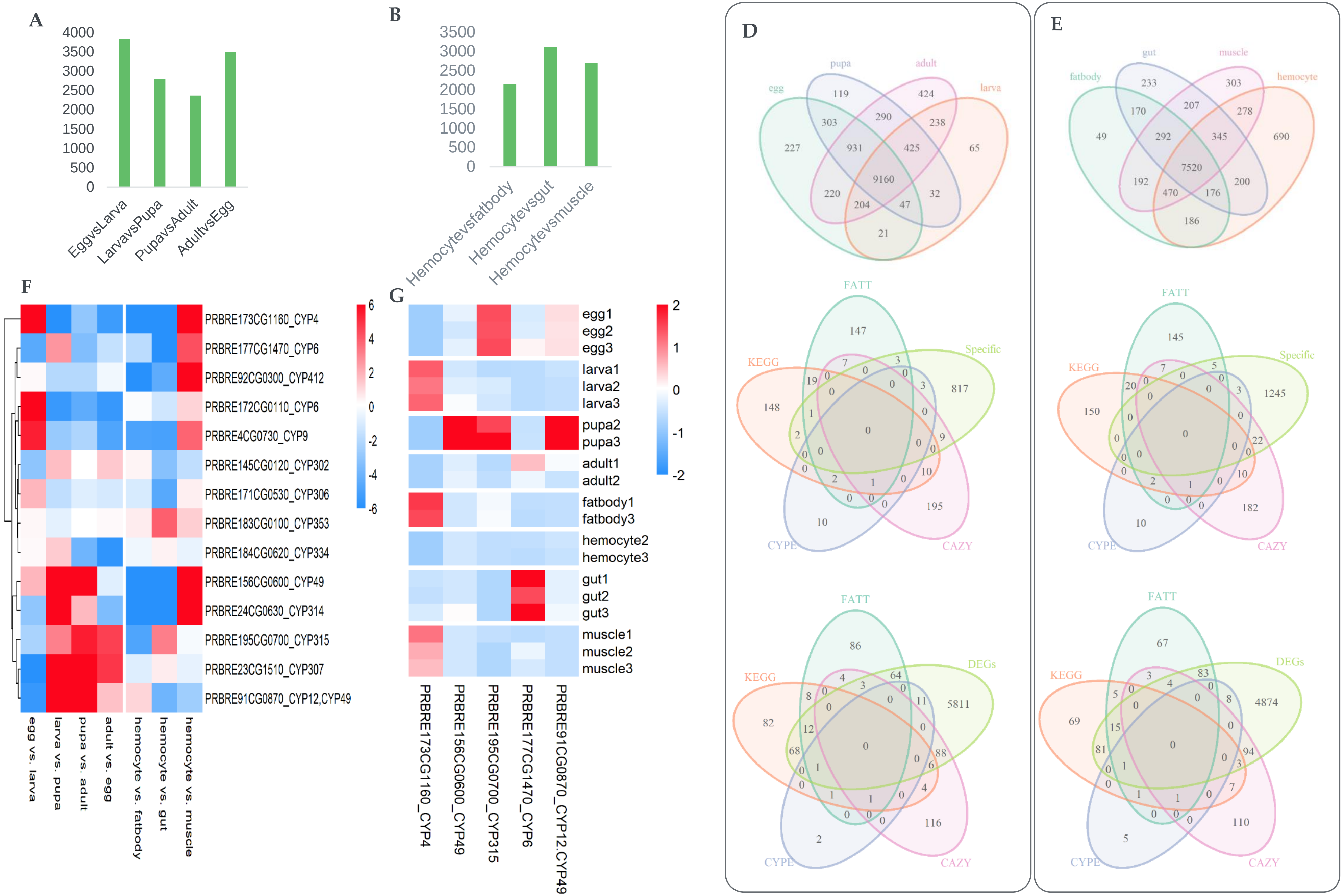

The overview of the RNA differential expression of two set of RNA seq samples, i.e., tissue specific and developmental stages of *Protaetia brevitarsis seulensis* insect. (The details parameters were given in PRBRE.geneInfo_RNA_Seq.xlsx file.

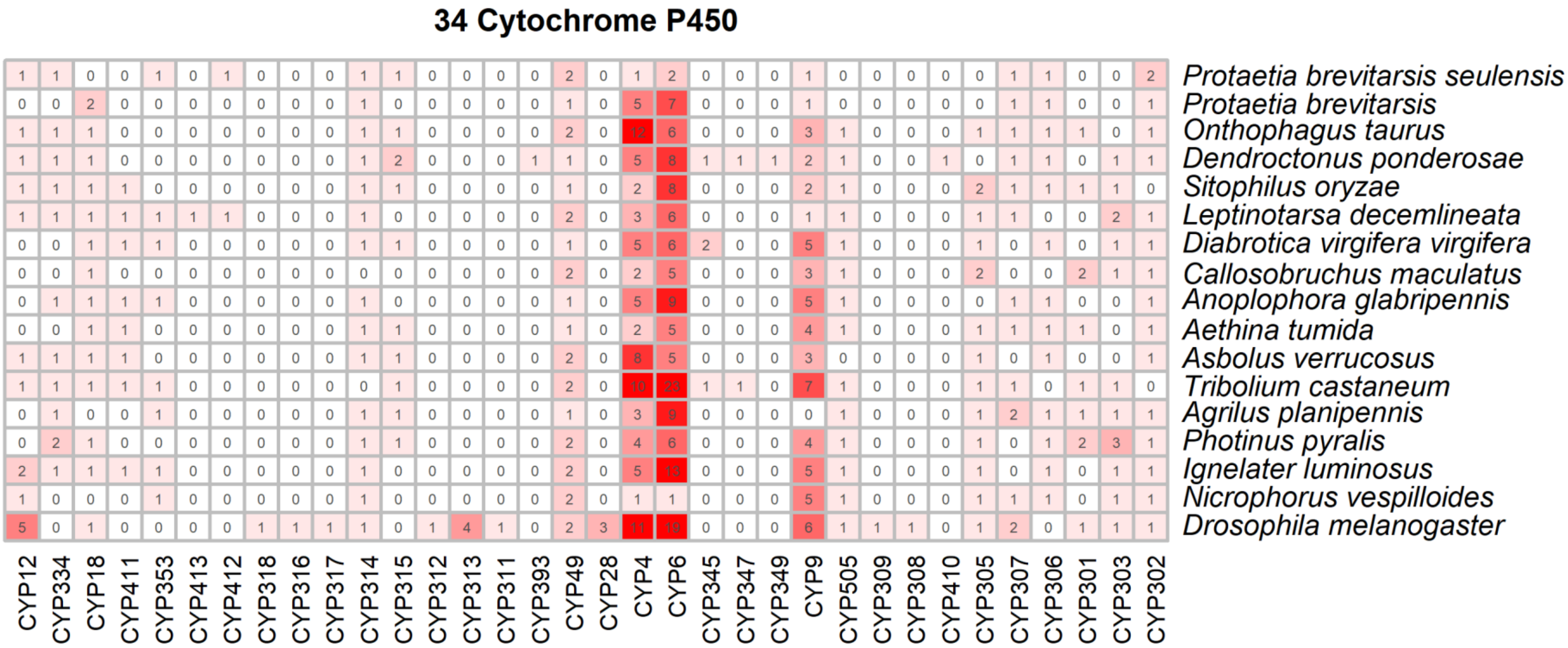

The overview of the cytochrome family genes present in the selected Coleoptera insect genomes.

SNO	Gene ID	CYPED	Annotation		Expression	
	PRBRE ID	Superfamily	Description	KEGG pathway	Expressed	DEG
1	PRBRE156CG0600	CYP49	probable cytochrome P450 49a1	.	O	O
2	PRBRE173CG1160	CYP4	cytochrome P450 4c3-like	.	O	O
3	PRBRE177CG1470	CYP6	cytochrome P450	.	O	O
4	PRBRE195CG0700	CYP315	cytochrome P450 315a1, mitochondrial	ko00981:Insect hormone biosynthesis	O	O
5	PRBRE91CG0870	CYP12,CYP49	probable cytochrome P450 49a1	.	O	O
6	PRBRE145CG0120	CYP302	cytochrome P450 302a1, mitochondrial	ko00981:Insect hormone biosynthesis		O
7	PRBRE171CG0530	CYP306	cytochrome P450 306a1	ko00981:Insect hormone biosynthesis		O
8	PRBRE172CG0110	CYP6	cytochrome P450	.		O
9	PRBRE183CG0100	CYP353	probable cytochrome P450 49a1	ko00100:Steroid biosynthesis;ko01100:Met abolic pathways;ko05152:Tuberculosis		O
10	PRBRE184CG0620	CYP334	probable cytochrome P450 49a1	.		O
11	PRBRE23CG1510	CYP307	cytochrome P450 307a1-like	ko00981:Insect hormone biosynthesis		O
12	PRBRE24CG0630	CYP314	ecdysone 20-monooxygenase	ko00981:Insect hormone biosynthesis		O
13	PRBRE4CG0730	CYP9	cytochrome P450 9e2-like	.		O
14	PRBRE92CG0300	CYP412	cytochrome P450 4d2-like	.		O

The overview of the cytochrome genes RNA differential expression of two set of RNA seq samples, i.e., tissue specific and developmental stages of *Protaetia brevitarsis seulensis* insect. (The details parameters were given in PRBRE.geneInfo_RNA_Seq.xlsx file.

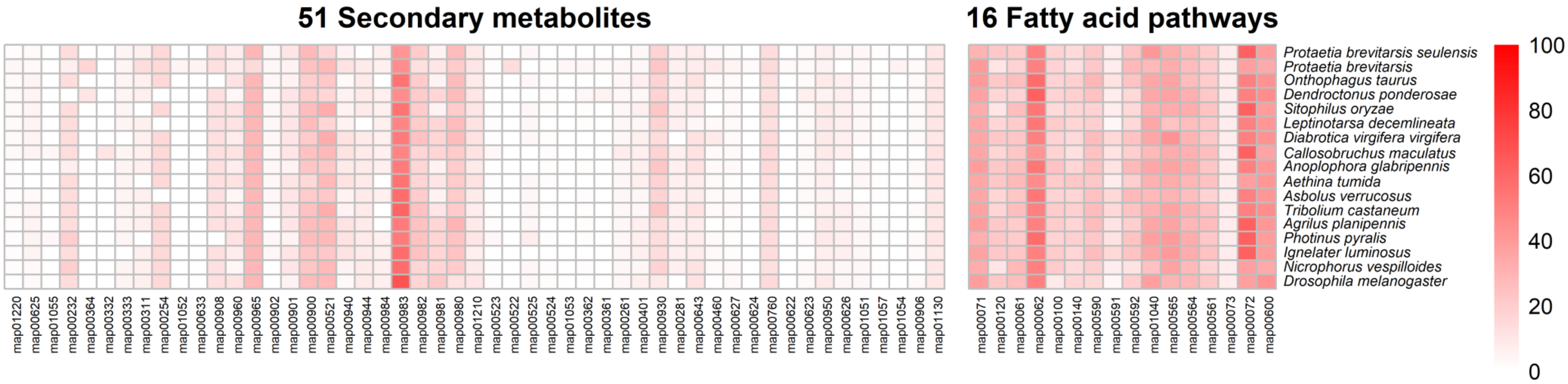

The overview of the KEGG secondary metabolites and fatty acid metabolism pathway genes present in the selected Coleoptera insect genomes.
